# Disparities in Lung Cancer Health Outcomes and Access to Lung Cancer Screening Between Rural and Urban Areas in the U.S

**DOI:** 10.3390/cancers18050864

**Published:** 2026-03-07

**Authors:** Aishani Gargapati, James Fox, Erminia Massarelli

**Affiliations:** 1 University of Texas at Tyler School of Medicine, Tyler, TX 75799, USA; agargapati@patriots.uttyler.edu (A.G.); james.fox@uttyler.edu (J.F.); 2 Department of Internal Medicine, University of Texas at Tyler School of Medicine, Tyler, TX 75799, USA

**Keywords:** rural, lung cancer, lung cancer screening, caregiver, United States

## Abstract

Lung cancer is one of the leading causes of mortality in the United States. Despite overall declines in incidence and mortality nationwide, rural communities continue to experience higher rates of lung cancer incidence and mortality than their urban counterparts, a disparity that has persisted over recent decades. This review synthesizes evidence from epidemiologic and clinical studies evaluating rural–urban differences in lung cancer incidence, mortality, diagnostic stage, access to screening, and treatment outcomes. Factors influencing these differences—tobacco use and environmental exposures, socioeconomic inequities, access to healthcare, and psychosocial and spiritual support—are examined as well. The review highlights the importance of increasing access to lung cancer screening and suggests interventions to improve early detection, access to treatment, and enhance psychosocial and spiritual support for patients and caregivers residing in rural areas. In this review, we have followed the urban–rural classification designated by the United States Census Bureau as a rural area consisting of populations, housing, and territory not included within an urban-classified area.

## Simple Summary

This narrative review aims to highlight disparities in health outcomes between rural and urban areas in the United States as well as psychosocial, environmental, medical, and policy-level factors influencing these disparities, while emphasizing differences in access to lung cancer screening between rural and urban areas. It provides further recommendations and interventions that can be implemented to increase access to lung cancer screening and care from the lens of the patient, caregiver, and healthcare provider.

## 1. Introduction

Lung cancer is estimated to be the leading cause of cancer mortality in the United States in 2026 and has represented a major contributor to health disparities across geographic regions [1,2]. Despite improvements in prevention, screening, and therapy in recent decades, the benefits of these advances have been inequitably distributed. It is estimated that 46.2 to 59 million individuals in the U.S. reside in rural areas [3], where the prevalence of smoking and lung cancer incidence in these areas are almost twice that of the largest metropolitan areas [4]. Lung cancer incidence and mortality have been shown to be higher in rural counties overall compared to urban counties [5,6]. Understanding the drivers of this geographic disparity is essential to enhancing the health outcomes of lung cancer patients residing in rural areas.

Although prior reviews have examined rural–urban disparities in lung cancer outcomes and factors influencing these disparities or application of specific artificial intelligence tools (AI) in lung cancer screening, these topics are often addressed in isolation. Our review synthesizes these topics comprehensively together. Furthermore, our review examines similarities and differences between rural and urban areas across the continuum of lung cancer—ranging from incidence and screening uptake to stage at diagnosis, treatment access, and survival.

In addition, this review highlights the importance of psychosocial and spiritual support for lung cancer patients and their caregivers —a topic less commonly discussed within the literature. Through this review, we look forward to highlighting the importance of this type of support and the essential role that caregivers play in the health of the care recipient in navigating lung cancer.

This review seeks to address the gaps above by (1) synthesizing evidence on rural–urban similarities and differences in lung cancer incidence, mortality, and stage at diagnosis; (2) examining disparities across the lung cancer care continuum, including screening, diagnosis, treatment, and follow-up; (3) evaluating the emerging role of AI-based screening tools and their potential implications in screening; (4) addressing the importance of psychosocial and spiritual support in lung cancer care; (5) and providing recommendations that could potentially enhance each component of the cancer care continuum.

## 2. Methods

A narrative literature review was conducted using searches of PubMed, Google Scholar, and JSTOR. The search included studies published from 1964 to present. Keywords were used in various combinations in searches, such as “lung cancer,” “rural,” “caregiver,” “screening,” and “artificial intelligence.” Eligible studies included original research articles, review articles, meta-analyses, retrospective and prospective studies, and epidemiological investigations published in peer-reviewed journals. Non-peer-reviewed articles were excluded. Articles were selected based on their relevance to the themes addressed in this review, including rural–urban disparities in lung cancer incidence and mortality; similarities and differences in stage at lung cancer diagnosis, treatment outcomes, and stage-specific mortality; factors influencing rural–urban disparities, such as tobacco use and environmental exposures, socioeconomic inequities, access to healthcare, and psychosocial and spiritual support; and applications of artificial intelligence to lung cancer screening. The selected literature was narratively synthesized and organized by theme. The review first provides an overview of rural–urban disparities in lung cancer incidence and mortality trends, followed by a discussion of disparities in screening, diagnosis, treatment, and survival, along with the underlying factors influencing these differences. Furthermore, it provides recommendations at the level of the community, medical practice, and policy that can increase access to lung cancer care and bridge rural–urban disparities.

## 3. Rural–Urban U.S. Trends in Lung Cancer Incidence and Mortality

### 3.1. Incidence Patterns

**Figure 1:**
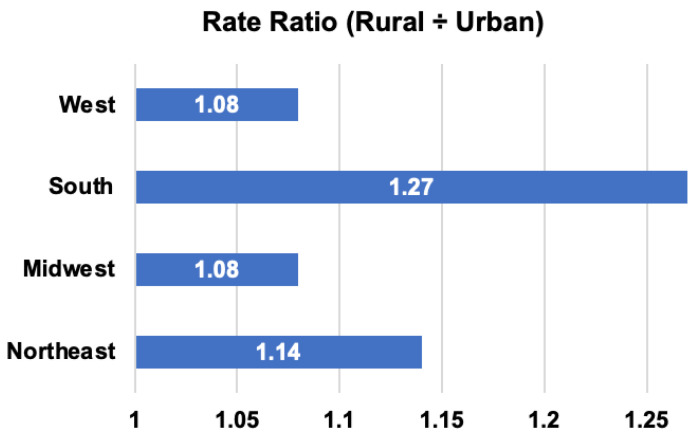
Rate ratios for age-adjusted lung cancer incidence rates by rural–urban status and U.S. Census Region, 2015–2019 [7].

National registry analyses demonstrate consistently higher lung cancer incidence in rural counties compared to urban regions [7]. It has been shown that from 2015 to 2019, rural areas had higher age-adjusted lung cancer incidence rates than urban areas across all U.S. regions, with the largest disparity observed in the South [7] (Figure 1).

The American Cancer Society estimates that in 2026, lung cancer will be the third-most common cancer diagnosis in men and women combined in the U.S. and second in new cases for both men and women, following prostate cancer in men and breast cancer in women [1]. Smoking continues to be the main risk factor, responsible for roughly 87% of cases in men and 84% in women. Although smoking rates have decreased significantly—from 42% of adults in 1964 to 11% in 2023—smoking drives the prevalence of most lung cancer cases in the country. About 2% of lung cancers occur before age 50, suggesting that this cancer type primarily affects older adults.

Various reports have been published regarding historical trends in lung cancer incidence among rural vs. urban areas. Howlader et al. reported that between 2001 and 2008, lung cancer incidence was similar across rural and urban U.S. areas among women of all races, but a clear divide emerged thereafter, with a 10% higher incidence in rural women by 2021 [7]. Among men, this disparity was interestingly consistent from 2001 to 2021—rural areas had about a 15% higher incidence in lung cancer than urban areas throughout these years. Since 1990, rural White and Black populations of both sexes have maintained consistently higher incidence rates than their urban counterparts, suggesting long-standing differences in exposure and cancer prevention efforts [7]. Moreover, while lung cancer incidence declined in both rural and urban counties from 1995 to 2013, this decrease was about three times faster in urban regions (−1.08% vs. −0.34% annual percent change), suggesting a slower uptake of lung cancer prevention, screening, and early detection interventions in rural settings [8].

Along with geographic variation, these patterns highlight how progress in reducing lung cancer risk has not been shared equally between rural and urban areas. Slower declines in rural lung cancer incidence may reflect delayed implementation of smoking cessation efforts, lower participation in screening programs, and gaps in public health communication, as will be discussed in the following sections.

### 3.2. Mortality Trends

Patterns of lung cancer mortality closely mirror those seen in incidence but have widened over time.

Historically, lung cancer mortality had been higher in urban areas during the mid-20 th century, reflecting earlier adoption of tobacco use and industrial exposures. However, as smoking rates declined more rapidly in cities, this pattern reversed. According to Singh et al., differences in lung cancer mortality between metropolitan and non-metropolitan areas had reversed and widened by the early 1990s, with mortality approximately 20% greater in rural areas by 2007 [9]. More recent data demonstrate that between 1999 and 2020, rural counties experienced both higher age-adjusted lung cancer mortality and slower declines in mortality than urban counties [4]. By 2020, lung cancer accounted for about 44% of all cancer deaths in rural counties, representing the most severe cancer type among these communities. Similarly, Blake et al. reported a 20.3% higher mortality rate in rural vs. metropolitan regions between 2009 and 2013 [10]. The shifting pattern of lung cancer mortality illustrates how progress in public health can be unevenly distributed across geography and culture. What was once a challenge seen in urban areas has over time become one greatly affecting the lives of many in rural areas. This reversal reflects not only differences in smoking trends but also the structural disparities seen in rural areas—where access to care is limited for many and medical advances arrive more slowly.

Lung cancer mortality rates are decreasing nationwide; however, this decline has been occurring more quickly in urban areas than in rural ones [1]. Urban residents benefit more from early detection and treatment advances, while rural areas are limited in access to low-dose CT (LDCT) screening and fewer specialized treatment centers. This gap in mortality rates seen between rural and urban areas is suggested in part due to lower participation in screening—in 2022, only 18% of eligible Americans underwent lung screening, with the lowest uptake among Native American populations [1].

Interestingly, rural patients diagnosed with Stage I non-small-cell lung cancer had shorter survival compared to their urban counterparts [4]. However, there was no significant difference found in the late stages of diseases (Stages II-IV) [4]. This disparity may be due to limited access to treatment for early-stage cancers in rural areas. However, advanced lung cancers have similarly poor survival outcomes regardless of geographic location.

Ethnic-specific analyses provide further insight. Rural White, American Indian/Alaska Native, and Asian/Pacific Islander women have been shown to experience 6–10% higher mortality than their urban counterparts, while disparities are less pronounced among Black and Hispanic women [10]. Furthermore, interestingly, from 1975 to 2011, there was not a significant increase in the 5-year survival rate for rural Hispanic patients. Whereas, the survival rate significantly increased from 57.2 to 59.0% for their urban counterparts [11]. These findings provides unique insight into how geography interconnects with culture, community, and lived experience in shaping lung cancer outcomes. Reducing the disparity seen between urban and rural areas calls for approaches rooted in the lifestyle and daily experiences of rural residents, where care is not only available but also accessible, culturally relevant, and sustained across generations.

## 4. Diagnostic Stage, Treatment Disparities, and Survival

### 4.1. Stage at Lung Cancer Diagnosis

Contrary to disparities in incidence and mortality between urban and rural areas, the stage of lung cancer at which patients are diagnosed does not differ markedly between rural and urban patients. According to a retrospective cohort study examining 348,002 patients diagnosed with lung cancer between 2000 and 2006, both rural and urban patients were found to learn about their lung cancer diagnosis around the same stage of disease progression, suggesting comparable access to initial evaluation or symptomatic presentation [4]. However, there may be significant differences in the management and treatment of lung cancer in its early stages that contribute to disparities in rates of mortality and incidence among rural vs. urban communities, as will be explained in the next section.

### 4.2. Treatment Disparities

Substantial variation exists in access to treatment and treatment outcomes among rural and urban areas.

Among patients with Stage I non-small-cell lung cancer (NSCLC), those residing in the most rural counties were significantly less likely to undergo surgical resection and exhibited a median survival 12 months shorter than urban patients [4]. Possible explanations include higher comorbidity burdens, fewer thoracic surgeons, and longer travel distances to specialized cancer centers. In addition, rural and urban residents with NSCLC who received treatment at urban institutions experienced higher-quality care and improved survival outcomes compared to rural residents treated at rural facilities [12]. This finding further highlights the persistent disparities in healthcare resources and access between urban and rural settings, suggesting that location of treatment may play a critical role in health outcomes for patients with lung cancer.

### 4.3. Stage-Specific Mortality

Mortality among patients with early-stage lung cancer is notably higher in rural areas compared with urban ones [4]. This difference likely reflects the challenges rural patients face in accessing timely and specialized care, including diagnostic services, surgical expertise, and follow-up treatment. However, once the disease reaches an advanced stage (Stages II–IV), mortality rates have been shown to be similar across rural and urban populations [4]. At the advanced stage, the uniformly poor prognosis and limited curative options for late-stage lung cancer appear to outweigh the impact of geographic differences in access to lung cancer care.

## 5. Major Factors Influencing Rural–Urban Disparities in Lung Cancer Health Outcomes

**Figure 2:**
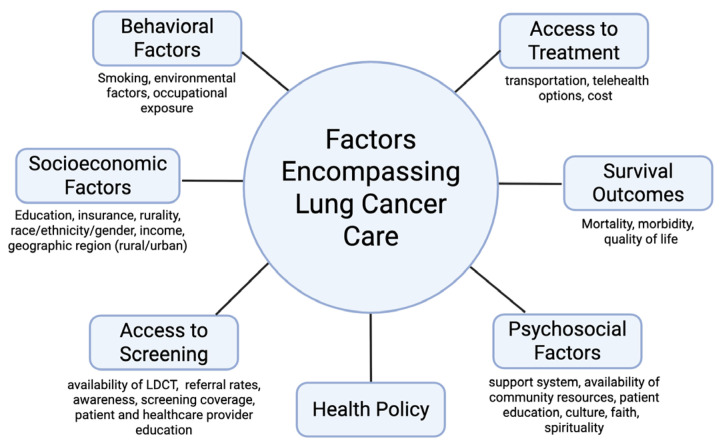
Factors Encompassing Lung Cancer Care.

Improving rural health outcomes from lung cancer requires addressing the unique challenges identified by the communities themselves. According to *Rural Healthy People 2030*, rural stakeholders rank mental health, addiction, and healthcare access and quality among their highest priorities, reflecting the deep connection between behavioral, social, and structural determinants of health [13]. These priorities also overlap with key factors known to shape disparities in lung cancer incidence and mortality. Issues, such as tobacco and substance use, limited access to healthcare services, socioeconomic disparities, and unmet psychosocial needs remain central to the rural health experience (Figure 2). Understanding how these elements interact with each other within a rural context is essential for developing targeted, equitable strategies to reduce the lung cancer burden. The following sections examine four major domains—tobacco use and environmental exposures, socioeconomic inequities, access to healthcare, as well as psychosocial and spiritual support—that together can help provide a framework in understanding and addressing rural–urban disparities in lung cancer outcomes.

### 5.1. Tobacco Use and Environmental Exposures

Higher smoking prevalence contributes greatly to the higher lung cancer incidence and mortality observed in rural populations. Rural residents are more likely to engage in smoking, initiate tobacco use earlier, and face barriers to cessation support, reflecting both behavioral and policy-level gaps [14]. According to a cross-sectional study of 303, 311 participants from the U.S. National Survey on Drug Use and Health from 2007 through 2014, smoking rates were shown to be consistently higher among rural adults, with current use reported at 30.1% for men and 24.8% for women, compared with 23.9% and 18.8% among urban men and women, respectively [15]. Moreover, declines in smoking prevalence have been slower in rural areas, widening the rural–urban gap since the mid-2000s, likely due to weaker tobacco control enforcement and fewer cessation programs. Rural residents are also more likely to use smokeless tobacco and occupy outdoor or manual labor positions, compounding their exposure to carcinogens and the risk of developing lung cancer [15].

In addition to tobacco use, environmental and occupational exposures further contribute to the observed disparities. Rural populations experience increased exposure to carcinogens through mining, agricultural work, diesel exhaust, and residential radon [16]. While urban residents face risks from vehicular emissions and industrial air pollution, studies have found that counties with high particulate-matter levels—often non-urban—exhibit significantly higher lung cancer mortality rates, along with lower income, education, and access to healthcare [17]. These findings underscore how behavioral, occupational, and environmental determinants influence lung cancer incidence and outcomes.

### 5.2. Socioeconomic Inequities

Socioeconomic conditions play a pivotal role in shaping observed differences in lung cancer incidence and mortality between rural and urban populations. Research using the Area Deprivation Index (ADI) demonstrates that lung cancer prevalence and mortality increase steadily with higher levels of socioeconomic deprivation, even after accounting for smoking, age, and sex [18]. In Maine, for instance, individuals residing in the most socioeconomically deprived areas had about 41% higher lung cancer prevalence and 59% higher mortality than those in the least deprived regions, underscoring how social and economic context influences lung cancer outcomes [18].

Broader analyses show that individuals from lower socioeconomic backgrounds are less likely to receive curative treatments, such as surgery or chemotherapy [19]. These findings highlight that social and structural factors, such as financial stability, educational attainment, and healthcare access—play a major role in shaping lung cancer trajectories.

Furthermore, rural populations often face unique socioeconomic challenges that intersect with healthcare delivery. Yet, these challenges also present opportunities to strengthen rural cancer care through targeted interventions, such as implementing mobile screening units, rural oncology networks for patients and their caregivers, and expanded telehealth services. As telemedicine grows, digital connectivity becomes increasingly important—although disparities persist, with non-metropolitan households, including rural ones, nearly twice as likely to lack reliable digital access [20]. Addressing this disparity can improve not only access to healthcare but also patient education and support.

Many rural patients demonstrate strong engagement and trust in their local providers, which can be leveraged to encourage them to participate in screening and engage more consistently in their care [21]. Understanding the unique experiences of rural residents can enable us to develop more personalized strategies to increase their access to healthcare and support them beyond the clinic.

### 5.3. Lung Cancer Screening, Diagnosis, and Care

Access to timely and comprehensive lung cancer screening and care additionally remains an important factor driving rural–urban disparities in incidence and mortality. Although rural residents bear a disproportionate burden of lung cancer risk, they often face structural barriers across the entire care continuum—from screening, diagnosis, treatment, to follow-up.

Lung cancer screening (LCS) with LDCT has proven effective in reducing mortality, yet participation remains low in rural areas [21,22]. Limited availability of accredited screening facilities, long travel distances, transportation barriers, and higher rates of uninsured individuals (12.3% vs. 10.1% in urban areas) contribute to reduced uptake [21]. As a result, there is a critical need to increase awareness about lung cancer screening, as well as promote its accessibility and infrastructure among these communities. Despite evidence demonstrating that screening with LDCT reduces lung cancer-specific mortality by approximately 20%, population-level uptake remains strikingly low—only 5–10% of eligible individuals have undergone screening about a decade after national screening recommendations were introduced in 2013 [23]. This gap is particularly concerning in rural regions.

Current data reveal substantial geographic inequalities in the prevalence and reach of LCS programs. According to an analysis conducted on the uptake of LCS in the U.S., screening uptake was 18% lower among rural residents compared with their urban counterparts, with significantly reduced access across every U.S. census region designated as rural [24]. This disparity persists while accounting for variations in lung cancer burden as well, suggesting that geographic accessibility and systemic barriers influence differences in LCS uptake between rural and urban areas. Supporting this, data from the National Health Interview Survey demonstrated that only 22.2% of rural residents lived within a 30 min drive of an American College of Radiology (ACR)- or Lung Cancer Alliance-designated screening center, compared to 83.2% of urban residents [25]. The limited number of rural radiology-related facilities renders screening to be a logistical and financial challenge for many high-risk individuals.

Furthermore, one of the most prominent challenges in rural settings is lack of public awareness and education about LCS, potentially due to fewer outreach efforts and community health campaigns as well as limited guidance from healthcare providers [23]. As a result, many individuals remain unaware that lung cancer screening is offered or there may be misunderstanding regarding eligibility criteria. Primary care providers—who are central to initiating screening—often cite insufficient time to communicate with patients regarding screening, limited knowledge of eligibility criteria, and insufficient local resources as barriers to recommending or coordinating LCS, limiting early detection efforts [23]. This knowledge gap not only reduces the likelihood that at-risk rural patients are referred for screening but also undermines the quality of the decision-making process itself. Shared decision-making is essential to ensure that patients and their caregivers are informed of the benefits and potential harms, such as false positives, overdiagnosis, and complications from follow-up procedures associated with screenings.

In addition, rural populations experience higher uninsured rates—12.3% compared with 10.1% in urban regions—and face additional barriers such as paying for screening, lack of paid time off to receive screening, and transportation difficulties associated with traveling long distances to metropolitan facilities [23]. At the policy level, lung cancer screening is not currently mandated for patients-at-risk. In the United States, the American Cancer Society and U.S Preventive Services Task Force (USPSTF) recommends annual screening for lung cancer with LDCT in adults ages 50 to 80 who have a 20 pack-year smoking history and currently smoke or have quit within the past 15 years [25]. Given the low levels of LCS uptake, further discussions can be made on mandating screening.

Furthermore, federal funding for lung cancer research remains lower than for other malignancies, despite the disease’s higher mortality burden. According to a study conducted on NIH funding data of cancer research from 2008 to 2023, lung cancer had the greatest disability-adjusted life years (DALYs) across all cancers studied—approximately 1200 DALYs per 100,000, and this trend was consistent across the course of the study. However, lung cancer research had the lowest ratio of funding to DALYs per 100,000 people (~$300,00) among the cancers, whereas neuroblastoma has the highest ratio of funding (~$14,000,000) [26]. Therefore, when adjusting funding relative to disease burden, lung cancer receives significantly lower research funding per unit of disease burden than many other cancers [26]. There could be potential reasons for why this may be, potentially due to greater emphasis on advocating for behavioral changes, such as decreasing smoking rates among communities, than on funding for advancing screening, care, and treatment. When examining cancer research funding from a resource-perspective, further federal funding can be allocated towards lung cancer research, which can be directed to enhancing the cancer care continuum. Addressing these policy-level challenges is essential to increasing access to lung cancer screening and care and developing interventions and methods identifying the disease at an early stage.

### 5.4. Recommendations for Improving Lung Cancer Screening, Diagnosis, and Care

Various strategies and interventions can be implemented to increase access and awareness of LCS in rural communities. One strategy can be to expand and simplify LCS eligibility criteria such that primary care providers can more easily and quickly identify eligible patients during routine care. Implementing electronic health record tools, such as best practice advisories, that flag eligible patients for screening could provide more opportunities for providers to begin discussing the topic of screening with patients and can streamline referrals. More hospitals and clinics are shifting to robust EMR systems, such as Epic, and many of these systems have these best practice advisories (BPAs) in place. However, the availability and functionality of BPAs may vary across EMR platforms, particularly in less robust systems. This could influence the consistency and effectiveness of these interventions across different settings of practice. Furthermore, there is growing evidence that screening eligibility could be revisited: it is shown that lung cancer risk continues to increase by 8.7% per year beyond 15 quit-years, implying that extending screening eligibility could prevent additional lung cancer deaths [27].

Another avenue to increase access to screening can be to embed mobile lung cancer screening programs within rural communities. Academic medical centers and community hospitals can develop partnerships with each other, where mobile CT units can be brought to community residents. While these partnerships are developing increasingly within a research context, outreach efforts for screening beyond research studies are limited and can be an area to further strengthen. In addition, logistical and operational challenges may limit practicality and scalability of establishing mobile CT units within rural communities. Along with academic-community partnerships, local health departments can launch educational campaigns about the risk factors of lung cancer and importance of screening and early detection. Engaging community leaders, churches, and veteran groups—particularly given that Veterans are three times more likely to meet USPSTF 2021 eligibility criteria and are more often located in rural areas—can enhance trust and participation [28].

In addition to expanding physical access, artificial intelligence (AI) imaging softwares, risk prediction tools, and computer-aided diagnostic (CAD) tools such as the EON patient management system can further augment LCS and holds transformative potential in addressing rural disparities in screening delivery. For instance, EON is a clinical management and screening tool that identifies eligible patients for lung cancer screening as per USPSTF criteria by scanning radiology reports using natural language processing to detect when lung nodules have been indicated in the report.

In addition to EON, it has been shown that AI-based tools can detect and classify pulmonary nodules on CT scans with sensitivity rates of 86–98%, outperforming radiologists in many studies, though with slightly lower specificity [29]. These systems can assist healthcare providers in interpreting scans in rural settings, where trained thoracic radiologists may be limited. Furthermore, AI could potentially assist in detecting lung cancer in its early stages rather than the late stages, as patients in the early stage of the disease may be asymptomatic or have few non-specific symptoms [30]. This can help rural clinicians more accurately detect lung cancer and can reduce diagnostic delays.

Recently, there have been exciting developments in AI that are being tested for their abilities to effectively screen and detect lung cancer. Deep learning algorithms, such as Google’s LDCT analysis model, the Sybil risk prediction system, and the Clinical Histopathology Imaging Evaluation Foundation, demonstrate how AI can enhance both detection and prognostication [31,32]. Google’s LDCT analysis model was tested using data from the National Lung Screening Trial. It correctly identified lung cancer cases with 94.4% accuracy and performed more strongly than six experienced radiologists. The tool also made 11% fewer false alarms and missed 5% fewer positive cancers, suggesting that AI can potentially improve accuracy and reliability in screening [31]. Similarly, the Sybil computer model could predict a patient’s future risk of lung cancer from one LDCT. It was very accurate, with a score of 0.92 for predicting cancer within one year and 0.75 for predicting cancer within six years, suggesting that this tool could assist clinicians in tracking a patient’s risk over time and decide how often they should get screened. In addition, researchers recently developed the Smart-LungNet lung classification tool, which is an enhanced form of the MobileNet V2 tool. The Smart-LungNet tool has been shown to effectively analyze chest X-ray images and classify them into three groups: Normal, Lung Opacity (areas that appear opacity or abnormal on X-ray), and Viral Pneumonia [32]. Among a dataset of 3475 chest X-ray images, the Smart-LungNet Tool classified lung conditions with 89.95% accuracy and outperformed prior tools tested on this dataset, such as ResNet18 and MobileNetV2 [32]. Integrating AI with biomarker-based screening could further individualize lung cancer detection. Studies, such as the Bio-MILD lung cancer screening trial, have demonstrated the utility of combining blood microRNA profiles with CT screening results to guide personalized screening intervals [33]. Thus, AI has great potential in aiding clinicians in timely identification of lung cancer.

However, there are potential limitations to reflect on when integrating AI into lung cancer screening and detection. As AI is trained upon its implementation in prior datasets, AI may not be as accurate in analyzing datasets that it has not trained previously on. These include datasets with differences in image quality, vendor platform, and scan conditions. For example, if an AI tool has previously analyzed data from urban hospitals primarily, its performance may not be as representative when used in a rural hospital [34]. Furthermore, AI models tend to underrepresent rural populations in training datasets [35], potentially due to these tools not being validated sufficiently in rural communiities [36]. Among 75,774 patients from The Society of Thoracic Surgeons General Thoracic Surgery database, it was shown that patients and those with private insurance had a higher incidence of receiving complex lung cancer operations [37]. This may be potentially due to screening models being trained more commonly among these populations. Along with potential bias in AI tools, AI tools tend to be limited in reproducing their results and do not clearly state the evidence-based features that led to its result [38]. Furthermore, it has been shown that AI-based LDCT screening tools have a sensitivity of 94.6% with a false-negative rate of 5.4% but only moderate specificity of 93.6% with a false-positive rate of 6.4% [39]. These findings suggest that these tools correctly identify 95 out of 100 true lung cancer cases. However, about 6 out of 100 people who are not diagnosed with cancer may be flagged as positive for cancer, and about 5 out of 100 true cancer cases may be missed. Therefore, considering the recent development of these tools and their potential errors, further rigorous evaluation of them would be required. Importantly, AI must complement—not replace—clinical expertise and judgement to ensure that interpretation from screening is fair and steps taken after screening are personalized to the patient.

Once patients at risk are diagnosed with lung cancer, rural patients frequently encounter additional challenges related to specialist availability. Nearly 40% of rural Americans with cancer report no cancer specialists near their communities [40]. Geographic analyses demonstrate that individuals living in counties with the least access to thoracic surgeons and radiation oncologists are significantly less likely to receive guideline-concordant treatment and experience higher mortality from early-stage non-small-cell lung cancer. These disparities are particularly pronounced among older, uninsured, and Medicaid-insured patients, as well as among Hispanic and Asian populations. Workforce shortages exacerbate these gaps: only 12.6% of U.S. radiation oncologists practice in rural areas, causing many patients to travel long distances for care.

Furthermore, medical centers located farther from remote areas receive referrals from these areas to conduct advanced diagnostic techniques for patients, due to their limited availability in these communities. Increasing access to diagnostic techniques in rural areas, such as robotic-assisted bronchoscopy and endobronchial ultrasound, to identify and analyze lung nodules in patients, is essential. These techniques have been shown to aid in minimally invasive diagnosis and staging and can help with identifying small lung nodules that may be more difficult to identify using conventional methods, such as transbronchial lung biopsy. Studies have shown that robotic-assisted bronchoscopy can accurately identify and safely biopsy small lung nodules [41], as well as aid in resecting small peripheral pulmonary nodules [42]. There is great potential for these tools to not only assist in LCS and diagnosis but also treatment. More resources and funding can be allocated to establish these technologies in rural areas.

Along with increasing access to LCS, ensuring that patients adhere to their screening timeline is critical. Establishing a centralized LCS program where one coordinated system or team manages most of the screening process can be a potential way to increase patient adherence. Ezenwankwo et al. conducted a meta-analysis on twelve studies, where they examined 17,195 patients who had initially negative LCS results and compared the adherence of these patients to the centralized or decentralized LCS program they were participating in [43]. They found that patients enrolled in centralized LCS programs were nearly twice as likely to return for their annual screening compared to those in decentralized programs—with adherence rates of 68.9% in centralized systems versus only 37.1% in decentralized ones [43]. Thus, centralized programs may reduce disparities in care and promote equity by making screening more organized and accessible to patients. In rural areas where a locally centralized program may not be feasible, a virtual centralized model could be developed with support from a nearby academic center or third party organization to coordinate LCS, similar to how telemedicine is structured.

Overall, increasing healthcare provider education about LCS and leveraging EHR tools, advanced diagnostics, and AI, the development of academic-community partnerships in the context of cancer care, and regional multidisciplinary networks can help bring healthcare expertise closer to rural communities (Table 1). Ensuring equitable access to lung cancer screening and treatment requires not only expanding the geographic reach of cancer services but also strengthening healthcare provider education and care coordination across rural health systems.

### 5.5. Psychosocial and Spiritual Support

Psychosocial and spiritual well-being are integral to comprehensive lung cancer care, yet they often receive less attention than social, structural, and economic factors. For rural residents, the experience of cancer care is shaped not only by geography but also by community, culture, and support systems. Patients frequently navigate long travel distances to access oncology services, often in challenging weather or with limited transportation options, which can contribute to physical and emotional fatigue [44]. These circumstances can also separate patients from familiar sources of comfort and social connection, especially when temporary housing near treatment centers may restrict family presence. Still, rural communities often show remarkable cohesion and mutual support, offering a strong foundation upon which more formal psychosocial and spiritual care resources can be built.

Qualitative studies highlight that many rural cancer survivors value the importance of peer connection but often lack structured opportunities to engage with others who share similar experiences [45]. Some express a preference for in-person interaction, while others find virtual or telehealth support groups a helpful alternative when travel may be challenging. Privacy and familiarity within small communities can occasionally make patients hesitant to discuss personal experiences openly, suggesting that flexible and confidential options for support are needed. Caregivers in rural settings similarly balance personal commitment with practical challenges such as travel, communication with healthcare providers, and financial strain [46,47,48].

Importantly, further exploration and attention to psychosocial and spiritual needs can meaningfully influence lung cancer treatment experiences and outcomes. At diagnosis, many patients often do not receive distress screening, especially if they are uninsured or reside in rural areas or areas with limited resources. In patients with lung cancer, emotional distress has been associated with family relationships consisting of emotional and physical strain, as well as limited information about diagnosis and treatment [49]. Learning about the emotional and physical needs of patients at the time when care is established and addressing these needs over the progress of care is essential to helping patients maintain a positive quality of life and wellbeing.

Caregivers are an integral pillar of support for patients as they provide care to them inside and outside of the clinic, coordinate their medical care with healthcare providers, and navigate with them together the emotional, physical, and financial challenges associated with lung cancer and treatment. It has been shown that there is an association between residing with a caregiver and lower reported supportive care needs in patients with advanced cancer [49]. Recognizing and strengthening the caregiver–patient dynamic can enhance the overall health and well-being of both patients and caregivers. In the clinic, healthcare providers can provide both patients and caregivers a questionnaire aimed to learn more about their wellbeing and needs such that they can best support them. In addition, providers can integrate faith and spirituality in their conversations with them when appropriate. Outside of the clinic, community-based support groups can be established to provide a space where patients and caregivers can share their feelings and experiences.

The findings above highlight the importance of further understanding the unique psychosocial and spiritual landscapes of rural communities to ensure that support interventions are not only available for rural residents, lung cancer patients, and caregivers but also are culturally resonant and inclusive.

## 6. Limitations of This Narrative Review

This narrative review has limitations that should be acknowledged. The scope of this review was focused on examining trends in lung cancer incidence and mortality in rural and urban areas beginning from the 1960s, as well as rural–urban differences across the continuum of lung cancer care from screening, diagnosis, treatment to survival outcomes. Additionally, this review examined the role of artificial intelligence in improving the accuracy and detection of lung cancer screening. As a narrative review, the search strategy was not fully systematic and did not follow a formal Preferred Reporting Items for Systematic Reviews and Meta-Analyses (PRISMA) protocol or include a structured risk-of-bias assessment. The literature search was derived primarily from PubMed, Google Scholar, and JSTOR, and relevant studies indexed in other databases such as Scopus or Web of Science may have been missed. Furthermore, heterogeneity in definitions of “rural” across studies—including the use of census classifications, RUCA codes, and metropolitan versus non-metropolitan designations—may influence the interpretation and synthesis of findings presented. Many of the included studies were observational, retrospective, registry-based, or ecological in design, which allowed for identifying associations but not causal conclusions. Finally, while this review highlights the growing application of artificial intelligence in lung cancer screening, the AI tools indicated have been recently developed. Thus, further validation would be required to assess the accuracy and precision of these tools among diverse populations.

## 7. Conclusions

Collectively, this review provides deeper insight into the trajectory of lung cancer health outcomes among rural vs. urban areas, as well as the major factors influencing these disparities. While overall lung cancer rates have been declining nationally, the decline is slower in rural areas compared to urban areas. The persistence of this disparity reflects a complex interplay between behavioral, psychosocial, socioeconomic, environmental, and systemic factors.

Reflecting on these factors, further steps can be taken to help address these disparities in lung cancer care and enhance each component of the care continuum. One step can be expanding access to lung cancer screening by increasing availability of LDCT screening in rural communities and integrating AI in LDCT image interpretation in areas with limited radiology expertise. Furthermore, more efforts at the level of the community and medical practice can be to provide more patient education about the negative health effects of tobacco use and the importance of an environmental-friendly space to limit exposure to smoke. Furthermore, there should be further efforts in monitoring the environment of residents in rural regions with agricultural and industrial activity. This can help prevent exposure to risk factors for developing lung cancer.

Moreover, integrating AI into clinicians’ workflow can help bridge shortages in cancer specialists and radiologists in rural areas. Furthermore, partnerships can be established between academic centers and rural health clinics to increase access to lung cancer screening technologies and cancer therapies that may be limited in rural areas. Likewise, recruiting more lung cancer specialists, radiologists, and healthcare team members to rural communities is essential. Beyond the structural barriers that rural communities experience, the psychosocial and spiritual dimensions of care profoundly influence the lived experience of lung cancer. Rural patient and caregiver support programs can be developed to support both patients and caregivers in navigating their journey. These programs can include providing virtual counseling, establishing peer-support networks, and integrating spiritual care into oncology services.

Research areas that can be further explored include similarities and differences among various demographics, such as race, gender, and ethnicity, throughout each stage of lung cancer care continuum. Furthermore, exploring models of lung cancer care currently implemented globally and in the U.S. in rural areas and how they compare and contrast would be deeply beneficial such that rural communities can potentially adapt these models in the future. Moreover, more studies validating AI tools across rural communities of diverse demographics can be conducted.

Addressing lung cancer disparities at the level of the individual, community, health system, and policy is essential to enhancing the health and wellbeing of rural communities and shaping rural lung cancer care into a model that is equitable, culturally grounded, and geographically responsive.

## Figures and Tables

**Table 1:** Rural–Urban Disparities Across the Lung Cancer Care Continuum.

Stage of Care	Key Rural–Urban Disparities	Contributing Factors	Potential Strategies
**Prevention**	Higher smoking prevalence and slower decline in lung cancer incidence in rural areas; greater exposure to radon and occupational carcinogens	Limited tobacco control and cessation programs; limited literacy on risk factors for lung cancer development; cultural normalization of smoking	Strengthen rural tobacco control policies; increase awareness about risk factors, increase accessibility to cessation services; improve environmental safety
**Screening**	Lower uptake of low-dose CT screening in rural areas; greater distances to travel to care facilities; higher rates of uninsured individuals	Limited screening infrastructure; transportation barriers; inadequate healthcare provider awareness about screening and patient outreach	Establish mobile screening units; augment screening with AI, CAD, robotic-assisted bronchoscopy, and endobronchial ultrasound; provider education on screening; provide transportation services for screenings
**Diagnosis**	More frequent late-stage presentation in rural areas; diagnostic and biopsy delays	Shortages of specialists and imaging facilities; limited coordination among healthcare providers regarding care and referrals	Recruit more cancer specialists; address social determinants of health and risk factors early in care to prevent further cancer progression
**Treatment**	Lower likelihood of surgical resection or receiving guideline-concordant therapy in rural areas; greater financial challenges	Specialist workforce shortages; rural hospital closures; travel and cost constraints	Expand oncology care network; establish more resources where patients and caregivers can receive further guidance and psychosocial support

## Data Availability

No new data were created or analyzed in this study. Data sharing is not applicable to this article.

## Abbreviations

The following abbreviations are used in this manuscript:

LCS	Lung Cancer Screening
LDCT	Low-dose Computed Tomography
ADI	Area Deprivation Index
USPSTF	United States Preventive Services Task Force
AI	Artificial Intelligence
BPAs	Best Practice Advisories
CAD	Computer-aided diagnostic
DALY	Disability-Adjusted Life Year
